# Energy-dense snacks can have the same expected satiation as sugar-containing beverages

**DOI:** 10.1016/j.appet.2015.06.007

**Published:** 2015-12-01

**Authors:** Ashley A. Martin, Liam R. Hamill, Sarah Davies, Peter J. Rogers, Jeffrey M. Brunstrom

**Affiliations:** Nutrition and Behaviour Unit, School of Experimental Psychology, University of Bristol, Bristol UK

**Keywords:** Sugar sweetened beverages, Satiety, Low energy sweetener, Viscosity, Learning, Expected satiation, Portion size, Texture

## Abstract

Sugar-sweetened beverages (SSBs) are thought to be problematic for weight management because energy delivered in liquid form may be less effective at suppressing appetite than solid foods. However, little is known about the relative ‘expected satiation’ (anticipated fullness) of SSBs and solid foods. This is relevant because expected satiation is an important determinant of portion selection and energy intake. Here, we used a method of constant stimuli to assess the expected satiation of test meals that were presented in combination with different caloric and non-caloric beverages (500 ml) (Experiment 1 and 2), as well as with high-energy solid snack foods (Experiment 2). All energy-containing beverages and snack foods were presented in 210 kcal portions. Both experiments found that expected satiation was greater for meals containing caloric versus non-caloric beverages (201.3 ± 17.3 vs. 185.4 ± 14.1 kcal in Experiment 2; *p* < 0.05). Further, Experiment 2 showed that this difference was greater in participants who were familiar with our test beverages, indicating a role for learning. Notably, we failed to observe a significant difference in expected satiation between any of the caloric beverages and snack foods in Experiment 2 (range: 192.5–205.2 kcal; *p* = 0.87). This finding suggests that it may be more appropriate to consider beverages and solid foods on the same continuum, recognizing that the expected satiation of some solid foods is as weak as some beverages.

## Highlights

• Sugar-sweetened beverages may promote less satiety than solid foods.
• However, it is unclear whether beverages differ from all solid foods.
• Here, expected satiation was similar for caloric beverages and solid snack foods.
• Concerns about energy-containing beverages should be interpreted in this context.

Consumption of caloric beverages has increased over the last 20 years in tandem with the rate of obesity (Bleich, Wang, Wang, & Gortmaker, 2009; Wang, Bleich, & Gortmaker, 2008). Recent estimates indicate that children and adults consume approximately 14–20% of their daily energy intake from beverages (Drewnowski, Rehm, & Constant, 2013; Ng, Mhurchu, Jebb, & Popkin, 2012; Slining & Popkin, 2013); Sugar-sweetened beverages (SSBs) have received particular attention, being associated with increased energy intake and weight gain (Fiorito, Marini, Francis, Smiciklas-Wright, & Birch, 2009; Malik, Pan, Willett, & Hu, 2013), in addition to metabolic perturbances (Bray & Popkin, 2013; Fagherazzi et al., 2013). Not surprisingly, it is now common advice to reduce or eliminate SSBs from one's diet (Hu, 2013; Pan et al., 2013; Services, 2010).

Part of the reason that SSBs are thought to contribute to weight gain is because beverages appear to suppress appetite and energy intake less than solid foods (de Graaf, 2011). Most of this evidence comes from preload studies that have examined the prandial and post-prandial responses of participants who consume caloric liquids versus sensory-matched, equicaloric semi/-solid foods. These studies show that liquids generate less satiation and satiety than higher-viscosity foods (Mattes, 2006; Mattes & Rothacker, 2001; Tsuchiya, Almiron-Roig, Lluch, Guyonnet, & Drewnowski, 2006; Wolf, Bray, & Popkin, 2008). Consistent with this observation, when offered access to otherwise identical liquid and semi-solid foods, low viscosity is associated with greater *ad libitum* intake (Hogenkamp, Mars, Stafleu, & de Graaf, 2012; Zijlstra, Mars, deWijk, Westerterp-Plantenga, & de Graaf, 2008) and poor energy compensation at a subsequent test meal (DiMeglio & Mattes, 2000; Flood-Obbagy & Rolls, 2009).

These findings have tended to promote a polarized position whereby liquids are assumed to always produce less satiety than solid foods. Consistent with this proposition, it has been suggested that liquids are inherently less satiating than solid foods because liquids are consumed more rapidly (de Wijk, Zijlstra, Mars, de Graaf, & Prinz, 2008; Zijlstra, Mars, De Wijk, Westerterp-Plantenga, & De Graaf, 2008) and pass more quickly through the gastrointestinal tract (Juvonen et al., 2009; Marciani et al., 2001; Zhu, Hsu, & Hollis, 2013) than solids, and because liquids are often consumed very rapidly, which limits the satiety that is generated by oral exposure (for reviews, see de Graaf, 2011; de Graaf & Kok, 2010; Hogenkamp & Schioth, 2013).

Others have cautioned that the difference between liquid and solid calories may be overstated, and that factors other than viscosity may be as important, if not more so, for determining the satiating properties of a food (e.g., Almiron-Roig, Chen, & Drewnowski, 2003; Almiron-Roig, Palla et al., 2013). Indeed, we suspect that comparisons between ‘liquid’ and ‘solid’ calories may be of limited benefit because this level of analysis fails to capture the large differences in satiety that are generated even across solid food forms (Brunstrom, Shakeshaft, & Scott-Samuel, 2008; Holt, BrandMiller, Petocz, & Farmakalidis, 1995). There are several solid foods that are thought to contribute to weight gain on the very basis that they fail to generate enough satiety (Blundell & Macdiarmid, 1997; Prentice & Jebb, 2003). Likewise, there are some liquids, such as soup, that generate excellent satiety responses (Flood & Rolls, 2007; Himaya & Louis-Sylvestre, 1998; Mattes, 2005).

Rather than asking whether beverages categorically deliver less satiation than solid foods, a more practical approach may be to evaluate the satiation of different beverages against the *continuum* of satiety responses that might otherwise be observed in a range of solid foods. Recent work from our group has used this approach to profile the ‘expected satiety’ of a variety of solid foods, documenting four to five fold differences in the amount of satiety these foods are expected to confer (Brunstrom et al., 2008). These expectations have been shown to be an excellent predictor of the number of calories individuals self-select and ultimately consume (Wilkinson et al., 2012). Moreover, this kind of meal planning appears to be extremely common (Fay et al., 2011).

We are aware of only a few studies that have examined the relationship between expected satiety and food form (i.e., liquid vs. solid). Hogenkamp et al. measured the expected satiation of custards that were presented in a liquid, semi-liquid, semi-solid, or solid form (Hogenkamp, Stafleu, Mars, Brunstrom, & de Graaf, 2011). They observed that participants' judgments of expected satiation increased with the thickness of the custard. This observation was replicated in a subsequent nutrient conditioning experiment (Hogenkamp et al., 2012). While these results demonstrate that the expected satiation of energy-containing liquids differs from sensory-matched solid foods, these two studies did not investigate the expected satiation of liquids consumed as beverages. This is an important distinction as consuming a liquid in the context of a ‘beverage’ versus a ‘food’ has been shown to impact its satiating effects (e.g., Mattes, 2005).

To our knowledge, only two studies have measured the expected satiation of commercially-available beverages. One of these explored the effects of liking, familiarity, and expected satiation on portion-size selection using a range of snack foods, including a bottle of SSB (Brogden & Almiron-Roig, 2010). Calorie-for-calorie, the SSB was expected to deliver the same amount of satiation as some solid snack foods (e.g., chocolate bar, muffin) and less satiation than others (e.g., crisps, ice cream). More recently, Almiron-Roig et al. examined participants' ability to judge the portion sizes of 33 different snacks and meals, including four caloric beverages (i.e., SSB, milk, orange juice, and hot chocolate) (Almiron-Roig, Solis-Trapala, Dodd, & Jebb, 2013). They observed that participants equally underestimated the number of standard portions that were contained in a range of low-to-medium energy-dense food items, regardless of whether the item was a snack, a mixed meal, or a beverage. Although neither of these studies investigated the relationship between food form and expected satiation explicitly, their results support our suspicion that the expected satiation of beverages does not always differ from solid foods.

There were three goals for the present study. The first was to explore whether people discriminate between non-caloric and caloric beverages when judging the expected satiation of a meal. This was accomplished in Experiment 1 using a computer-based task that was designed to assess the expected satiation of meals presented in combination with a sugar-sweetened beverage (e.g., SSB), a low-energy sweetener beverage (LES), or water. This effect was replicated in Experiment 2 with a design which also allowed us to establish the relative contribution of calories versus carbonation to the expected satiation of these beverages. Our second objective was to compare the expected satiation of beverages relative to two solid foods. This was accomplished in Experiment 2, where we repeated our measures of the different beverage meals and compared these conditions with meals in which the beverage was replaced with a portion of solid snack food that was equicaloric to the calorie-containing beverages (210 kcals).

Our third objective was to explore individual differences in our participants' judgments of expected satiation. Previous research has shown that familiarity is a strong predictor of expected satiety (e.g., Brunstrom, Shakeshaft, & Alexander, 2010). There is also some evidence that the impact of a sweetened beverage on appetite is dependent on whether an individual typically consumes non/-caloric versions of that beverage (Appleton & Blundell, 2007; Appleton, Rogers, & Blundell, 2004). For these reasons, we hypothesized that individuals who frequently consumed SSBs might be more familiar with their satiating properties and, thus, more likely to discriminate between SSBs and non-caloric beverages (LES or water). This prediction was tested in Experiments 1 and 2 by examining the relationships between participants' intakes of SSBs and LESs, and their judgments of expected satiation.

## 1 Experiment 1

### 1.1 Method

#### 1.1.1 Participants

Sixty-eight undergraduates from the University of Bristol (UK) participated in the experiment for class credit (60 F/8 M; Age: *M* = 19.5, *SD* = 1.7). Their BMI ranged from 15.7 to 31.0 (*M* = 21.5, *SD* = 2.8). Current dieters and individuals taking a medication (other than contraceptive pills) that might influence appetite were excluded. Ethical approval was obtained from the local Faculty of Science Human Research Ethics Committee.

#### 1.1.2 Materials

##### 1.1.2.1 Test stimuli

Participants evaluated the expected satiation of two reference meals, each of which consisted of a savoury snack and a chocolate bar. One meal comprised a 32.5 g bag of salted potato chips (Walkers, Leicester, England) and a Mars^®^ bar (i.e., chocolate-covered nougat; Mars Incorporated UK, Slough, England) (total energy content: 431 kcals). The other meal comprised a 100 g bag of salted peanuts and a Twix^®^ bar (i.e., chocolate-covered biscuit with caramel; Mars Incorporated UK, Slough, England) (total energy content: 869 kcals). These snack items were selected because they are widely available and commonly consumed throughout the UK.

In this study, we elected to use two reference meals in order to generate greater variety across trials and to reduce participant fatigue. The critical manipulation was that each of these reference meals was presented in compound with three different beverages: a SSB (Coca-Cola), a LES (Diet Coke), and a matched volume (500 ml) of water; this yielded six possible meal–beverage combinations (hereafter referred to as ‘test meals’). By contrasting participants' judgments of meals that were identical in all respects except for the beverage, we were able to assess the relative contribution of different beverages to the expected satiation of a meal.

##### 1.1.2.2 Measurement of expected satiation

**Fig. 1:**
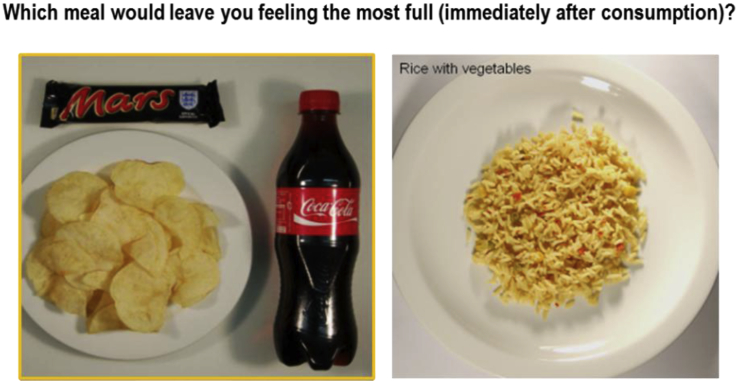
Schematic representation of the expected satiation task. On each trial, participants were instructed to imagine consuming each portion of food and to indicate which meal they believed would leave them feeling “the most full immediately after consumption”.

A Method of Constant Stimuli (for details, see Brunstrom et al., 2008) was used to examine how different beverages contribute to the expected satiation of a meal. Briefly, participants completed a series of trials in which they were asked to choose between two different meals—a test meal (see ‘Test stimuli’, above) or a portion of rice and vegetables (Uncle Ben's^®^, Mars Incorporated UK, Slough, England; energy density = 1.5 kcal/g) (Fig. 1). On each trial, a picture of one of the test meals was presented next to a picture of a portion of rice that ranged from 10 kcal–1000 kcal. Participants were asked to imagine eating each of the two meals and to indicate, by pressing the left or right arrow key, which meal would leave them feeling “the most full immediately after consumption”.

Participants completed 56 trials per test meal in order to derive a point of subjective equality (PSE) between the test meal and the comparison meal of rice and vegetables. This PSE represents the amount of rice (in kcal) that is expected to deliver the same satiation as the test meal. To derive a single PSE for each beverage condition, the PSEs for the test meals were averaged according to beverage type (SSB, LES, and Water). Thus, for each participant, there were three PSEs which corresponded to the average satiation of a meal paired with SSB vs. LES vs. Water. These PSEs are hereafter referred to as expected satiation values.

##### 1.1.2.3 Beverage intake

A questionnaire was used to assess how often participants consumed Coca-Cola, Diet Coke, ‘other SSBs’, and ‘other LESs’. Participants selected one of the following options indicating their intake: “Every day”; “2–3 times per week”; “Once a week”; “1–3 times per month”; or “Less than once a month”. For data analysis, the responses to the questionnaire were converted to frequency scores (“Every Day” = 5; “Less than once a month” = 1). These frequency scores were then summed (i.e., ‘Coca Cola’ + ‘other SSBs’; ‘Diet Coke’ + ‘other LESs’) to provide a single ‘familiarity score,’ reflecting each participant's typical intake of SSBs and LESs.

#### 1.1.3 Procedure

Participants were tested between 10:00–17:00 on weekdays. On arrival, they provided written consent and were given detailed instructions for completing the expected satiation task. In particular, the participants were instructed to actively imagine eating each of the meals on each trial in order to help them choose which of the two meals would leave them feeling the ‘most full immediately after eating’. After completing the expected satiation task, participants were issued the beverage questionnaire and their height and weight was measured.

#### 1.1.4 Statistical analysis

Expected satiation values were analyzed using repeated-measures ANOVA with Beverage (Water, LES, SSB) entered as a within-subjects factor to determine whether the participants discriminated between non/-caloric beverages when estimating the satiation of a meal. To explore whether the expected satiation of different beverages depended on beverage familiarity, we calculated correlation coefficients to assess the relationship between the expected satiation values of meals that were paired with LES and SSB and self-reported intakes of these two beverages. In addition, a difference score was calculated between the expected satiation values of the SSB and LES meals (SSB minus LES). This difference score provides a specific measure of the participants' ability to discriminate between the non/-caloric beverages, with positive scores indicating that meals containing a SSB were expected to deliver more satiation than the same meals paired with an LES. We calculated correlation coefficients to assess the relationship between these difference scores and our two familiarity scores (i.e., familiarity with SSB and LES, separately). These correlation coefficients were used to assess whether familiarity with either the SSB or the LES is associated with a greater difference in their expected satiation. Effect size was assessed for the repeated measures analyses using partial eta-squared ($n_{p}^{2}$), where a small effect is ∼0.02, a medium effect ∼ 0.13, and a large effect ∼ 0.26 (Cohen, 1988).

## 2 Results

**Fig. 2:**
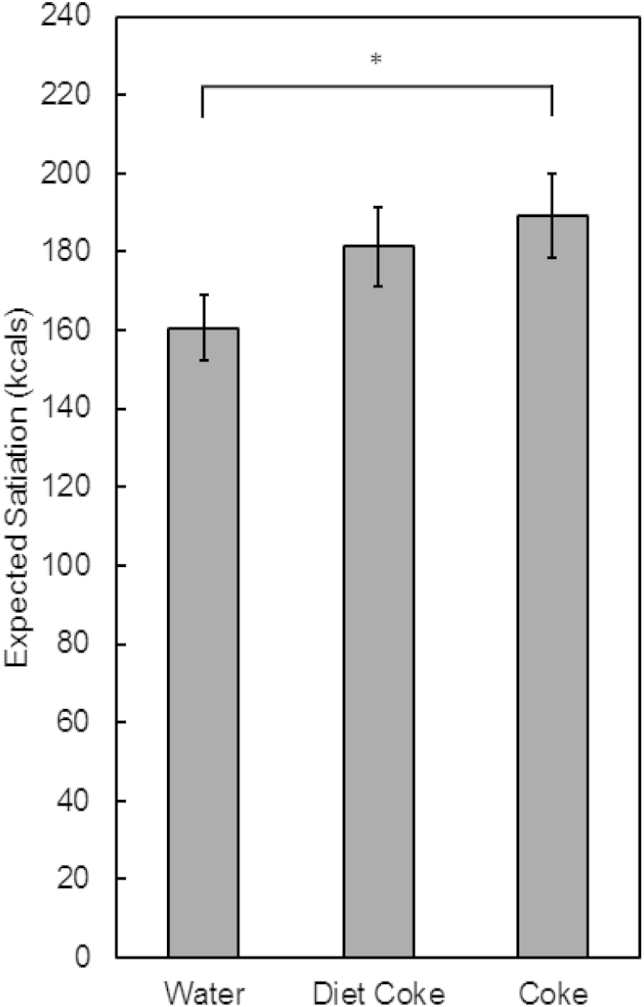
Expected satiation of test meals containing water, Diet Coke (LES), or Coca-Cola (SSB). Meals containing Coca-Cola were perceived to be more satiating than meals containing water. All other contrasts were non significant. *p < 0.05, *post-hoc* Newman–Keuls test.

As shown in Fig. 2, participants expected greater satiation from meals containing a SSB than meals containing an LES or water. This result was confirmed by a significant main effect of beverage (F(2, 134) = 16.95, *p* < 0.00001, $n_{p}^{2}$ = 0.20). *Post-hoc* Newman–Keuls tests indicated that, compared to water, meals containing an SSB (*p* = 0.00002) or LES (*p* = 0.00005) were expected to be more satiating. However, the expected satiation of the SSB was not significantly greater than the expected satiation of the LES (*p* = 0.12). There was little evidence that the expected satiation of the meals containing LES or SSB was related to the frequency with which these beverages were consumed (largest *r* = −0.18, all *p* > 0.05).

## 3 Experiment 2

**Fig. 3:**
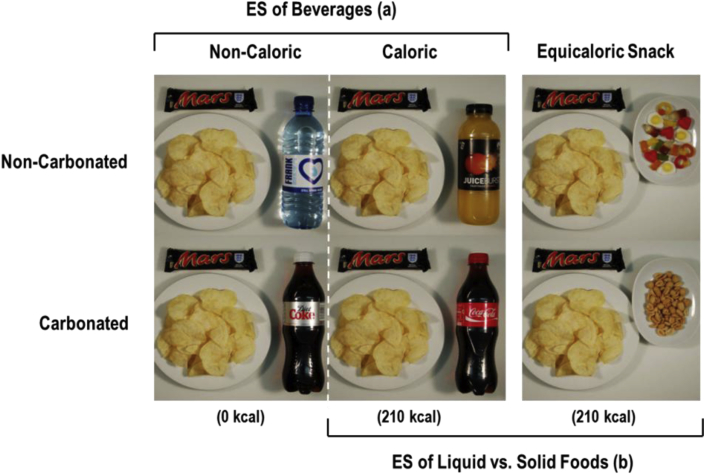
Schematic presentation of comparisons made in Experiment 2. (a) Expected satiation (ES) of test meals that contained non/-caloric beverages that were either carbonated (Diet Coke vs. Coca-Cola) or non-carbonated (Water vs. Orange Juice) were compared in Experiment 1. (b) An additional two test meals were included in Experiment 2 wherein the 210 kcal caloric beverage was replaced with a 210 kcal portion of snack food to compare the expected satiation of liquid versus solid calories.

Experiment 1 provided preliminary evidence that individuals discriminate between different types of beverages when judging the expected satiation of a meal. Experiment 2 was designed to determine whether this result can be replicated and to establish whether the expected satiation of a caloric beverage differs from equivalent portions (210 kcal) of a solid snack food. In addition, we reasoned that carbonation might have contributed to the observed difference between the still water and the SSB in Experiment 1 (e.g., Moorhead, Livingstone, Dunne, & Welch, 2008). Therefore, we included an assessment of the effects of a non-carbonated energy-containing beverage (orange juice). This enabled us to assess the relative and independent contribution of calories and carbonation to expected satiation (see Fig. 3).

## 4 Method

### 4.1 Participants

Eighty individuals were recruited from the University of Bristol (UK) and surrounding community to participate in the experiment. Participants were predominantly normal-weight women (52 F/28 M; Age: *M* = 22.2, *SD* = 6.7) with a BMI ranging from 16.8 to 31.3 (*M* = 22.5, *SD* = 3.0). Current dieters and individuals taking medications (other than contraceptive pills) which could influence appetite were excluded from participating. Participants received either £5 Sterling or class credits for their assistance. Ethical approval was obtained from the local Faculty of Science Human Research Ethics Committee.

### 4.2 Materials

#### 4.2.1 Test stimuli

As in Experiment 1, the reference meals consisted of a savoury snack and a chocolate bar. However, we replaced the peanuts with Hula Hoops^®^ (a potato-based snack food that is popular in the UK; KP Snacks, Leicestershire, UK) so that we could use the peanuts as a comparison food for the beverage conditions. This also allowed us to better equate the energy/macronutrient content of the two reference meals (Walkers Crisps + Mars bar = 431 total kcals; Hula Hoops + Twix bar = 422 total kcals).

#### 4.2.2 Measurement of expected satiation

Expected satiation was assessed using the same task described in Experiment 1. Each of the two reference meals was presented alongside four beverages (Coca-Cola, Diet Coke, Orange Juice, Water) (see Fig. 3), as well as two snack foods that were presented in a portion size that was equicaloric to the caloric beverages (33.9 g of salted peanuts; 61 g of Haribo Starmix gummy sweets). The beverages were all presented in 500 ml portions, with the energy content matched between the two caloric beverages (Coca-Cola and Orange Juice were each 210 kcals). This yielded 12 test meals in total. Participants completed 56 trials for each test meal (672 trials, total). As in Experiment 1, these test meals were averaged to yield one PSE corresponding to each beverage/snack food condition (6 total).

#### 4.2.3 Beverage intake and diet history

Intake of each of the test foods was assessed with a modified version of the beverage questionnaire from Experiment 1. In addition to assessing intake of SSBs and LESs, the questionnaire was broadened to include other non/-carbonated sweet drinks (i.e., squash, Ribena^®^ - a fruit-based drink similar to grape juice that is popular in the UK), 100% fruit juices, as well as the two solid snack foods used in the present study (peanuts and Haribo^®^). Each participant's frequency of consuming these items was calculated as described in Experiment 1. Liking was assessed using a seven-point Likert scale (How much do you like [the test food]? Anchors: Not at all – Extremely). All of the test food stimuli were generally liked (M = 3.4–5.8) by the participants.

### 4.3 Procedure

Testing took place between 10:00–17:00 on weekdays. On arrival, participants provided written consent and completed baseline appetite ratings. Baseline hunger, fullness, and thirst were assessed with the question, ‘How hungry/full/thirsty do you feel right now?’ with participants indicating their answer on a 7-point Likert scale (Anchors: Not at all – Extremely). The participants were then given detailed instructions for completing the expected satiation task. After completing the task, participants' liking and familiarity with the test foods (i.e., beverages and matched snack foods) was assessed and their height and weight was measured.

### 4.4 Statistical analysis

Expected satiation of the four beverage conditions were analyzed by repeated-measures ANOVA with Carbonation (Non/-Carbonated) and Calories (Non/-Caloric) as within-subjects factors. This allowed us to assess the independent and additive contribution of calories and carbonation to the expected satiation of beverages. A separate ANOVA with Item as a factor (Coca-Cola, Orange Juice, Peanuts, Haribo) was used to compare the expected satiation of the meals that included a caloric beverage with the expected satiation of meals that included an equicaloric portion of solid food. This allowed us to determine whether the sugar-containing beverages were expected to deliver less satiation than the energy-matched solid snack foods.

In order to explore whether dietary experience influences judgments of expected satiation, we collected each participant's frequency of consuming sugar-containing beverages (i.e., SSBs, juices, and non-carbonated beverages, like squash) and non-caloric sweetened beverages (i.e., LESs and non-carbonated beverages, like sugar-free squash). These two familiarity scores were used to calculate correlation coefficients to estimate associations with the expected satiation of these test meals. This enabled us to determine whether intake of the different beverages predicted participants' estimates of their satiation.

Two difference scores were also included in this correlational analysis: 1) the difference between the expected satiation of meals containing non-caloric vs. caloric beverages (Caloric minus Non-Caloric), and 2) the difference in expected satiation between meals containing caloric beverages vs. equicaloric solid foods (Solid minus Liquid). These difference scores were used to specifically assess whether people expect different outcomes from beverages depending on whether they are caloric or non-caloric (1), and whether people expect different outcomes from calories delivered in a liquid or solid form (2).

## 5 Results

Appetite ratings collected at the start of the session indicated that participants were moderately full at the time of testing (hunger: *M* = 3.3, *SD* = 1.74; fullness: *M* = 4.1, *SD* = 1.8; thirst: *M* = 4.1, *SD* = 1.3). To rule out the possibility that the differences in expected satiation between SSB and Water observed in Experiment 1 were attributable to differences in carbonation rather than energy content, a repeated-measures ANOVA was conducted.

**Fig. 4:**
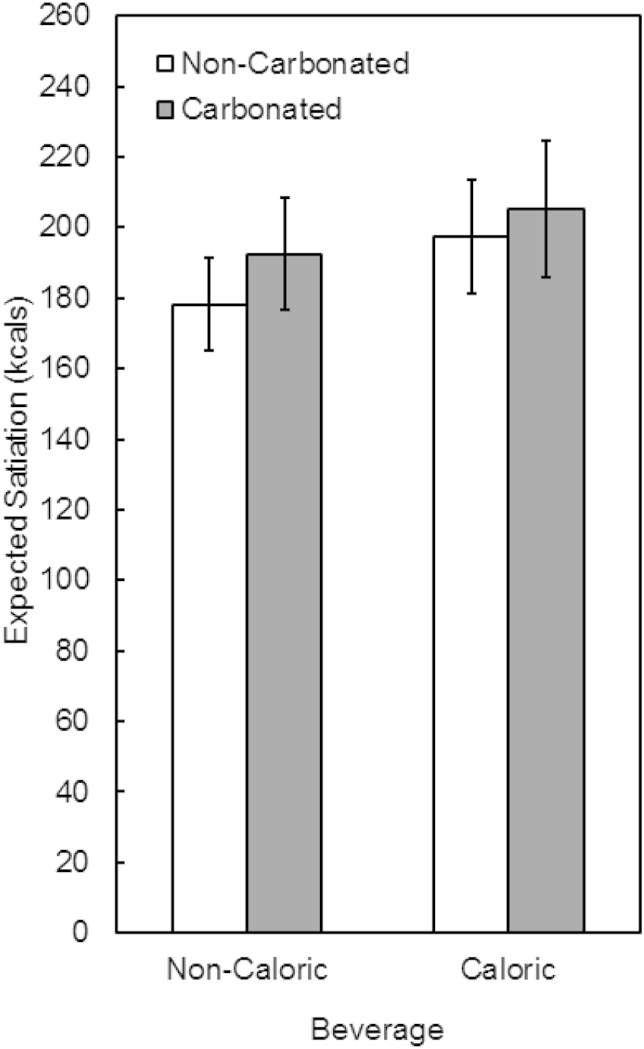
Expected satiation of meals containing caloric or non-caloric beverages. Meals containing a caloric beverage were expected to be more satiating than meals containing a non-caloric beverage. Carbonation did not significantly impact the expected satiation of the beverages.

As shown in Fig. 4, results confirmed that participants expected greater satiation from the meal when it contained a caloric beverage than a non-caloric beverage (201.3 ± 17.3 vs. 185.4 ± 14.1 kcal, respectively), and this effect did not depend on whether the beverages were carbonated or not. This pattern of results was supported by a significant main effect of Calories (F(1, 79) = 7.05, *p* = 0.01, $n_{p}^{2}$ = 0.08) and a non-significant Calories × Carbonation interaction (F(1, 79) = 0.40, *p* = 0.53, *η*_*p*_^*2*^ = 0.005). There was a trend for participants to expect the carbonated beverages to deliver more satiation than the non-carbonated beverages (198.82 ± 17.2 vs. 187.84 ± 14.4 kcal, respectively). However, this effect did not reach significance (main effect of Carbonation, F(1, 79) = 2.56, *p* = 0.11, $n_{p}^{2}$ = 0.03).

**Fig. 5:**
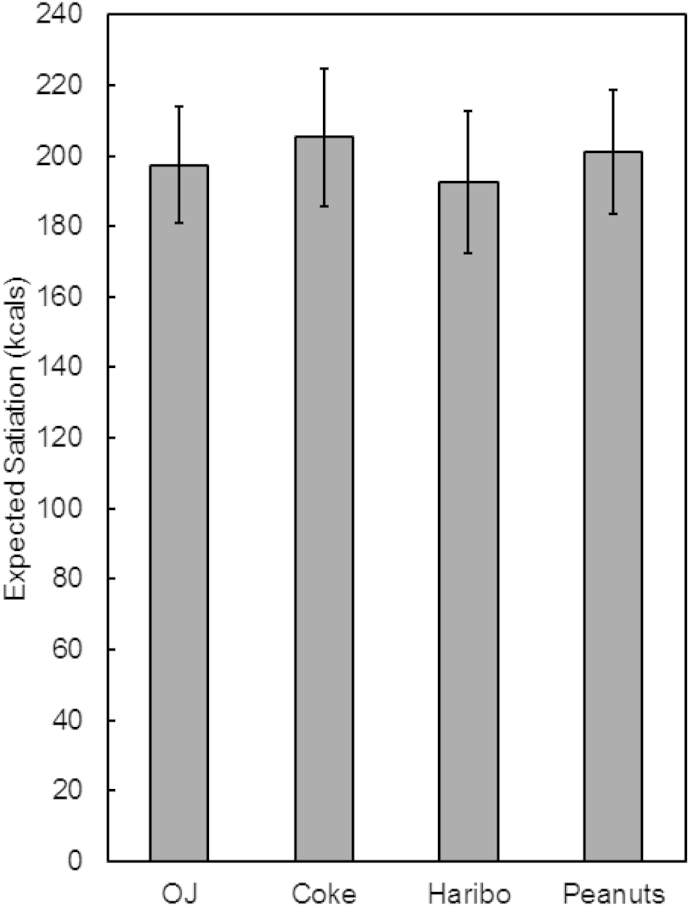
Expected satiation of meals containing a 210 kcal beverage (orange juice, Coca-Cola) or a 210 kcal portion of snack food (Haribo, Peanuts). Meals containing caloric beverages were expected to deliver the same amount of satiation as meals containing an equicaloric portion of solid food.

In order to determine the effects of food form on expected satiation, we conducted a separate ANOVA comparing the meals that contained either a 210-kcal beverage or a 210-kcal portion of solid snack food. As shown in Fig. 5, participants expected similar levels of satiation from these meals, regardless of whether they contained a beverage or a solid snack food (main effect of Item, F(3, 237) = 0.24, *p* = 0.87, $n_{p}^{2}$ = 0.003).

Lastly, we explored whether participants' expectations about non-caloric and caloric liquids and solid foods depended upon how frequently they consumed these foods in everyday life. As shown in Table 1, higher consumption of sugar-containing beverages (i.e., SSB + Juice intake) was associated with greater expected satiation of the meals containing Coca-Cola (*r* = 0.27), as well as a larger magnitude of discrimination between non-caloric and caloric beverages (Caloric minus Non-caloric; *r* = 0.38). A similar pattern of results was observed for LESs, with greater intake predicting greater expected satiation from the meals containing a SSB (*r* = 0.28) and greater magnitude of discrimination between non/-caloric beverages (*r* = 0.42).

One explanation for the counterintuitive observation that greater intake of LESs was related to an enhanced expected satiation of SSBs is that participants who frequently consumed LESs also tended to consume high quantities of sugar-containing soft drinks. Indeed*, post-hoc* correlations confirmed that intake of LESs was significantly correlated with intake of SSBs (*r* = 0.30). Higher intakes of both LESs and SSBs did not significantly impact participants' differentiation between calories from beverages versus snack foods (i.e., Solid minus Liquid scores).

## 6 General discussion

Our results indicate that people alter their expectations of how satiating a meal will be depending on the type of beverage that accompanies it, and that solid foods are not always expected to deliver greater satiation than beverages. Indeed, on a calorie-for-calorie basis, we found that some high-energy snack foods were expected to deliver the same level of satiation as a sugar-containing beverage.

On the basis that liquids receive less oral exposure and post-oral processing, one might expect an individual to learn to expect less satiation from liquids than solid foods (e.g., Cassady, Considine, & Mattes, 2012; Chambers, Ells, & Yeomans, 2013; McCrickerd, Chambers, Brunstrom, & Yeomans, 2012; Yeomans & Chambers, 2011)—so why were beverages not expected to be less satiating than solid foods, here? We believe it is because there is overlap between the range of satiety responses produced by liquid and solid foods that, until now, has been largely unexplored.

Consistent with this idea, our data demonstrate that sugar-containing beverages do not differ from a selection of *high-energy* solid snack foods. However, it is likely that robust differences would be observed if we contrasted our beverages with a selection of *low-energy* solid foods. It is in this regard that beverages appear to be unique—whereas low energy-dense foods will typically promote greater satiation than high energy-dense foods (due to their greater volumes) (e.g., Williams, Roe, & Rolls, 2014), this ‘rule’ appears not to apply for beverages. As seen in our own results, an equal-caloric beverage was not expected to generate more satiation than a solid snack food despite being eight times greater in volume.

Although the caloric beverages were expected to contribute to the overall satiation from the test meals, the effect was relatively small—representing an increase of approximately 20 kcals of rice compared to the non-caloric beverage conditions. At face value, this difference appears modest relative to the actual difference in energy content between these test meals (i.e., 210 kcals). However, the participants were never explicitly asked to provide an estimate of how many calories were in the meals or the test stimuli. The fact that a 430–630 kcal test meal is judged as delivering the same amount of satiation as a 160–220 kcal portion of rice simply demonstrates what is already known– that high-fat, high-energy foods (i.e., the crisps and chocolate in the test meal) are expected to deliver less satiation than low-fat, low energy foods (i.e., the portion of rice) (e.g., Brunstrom et al., 2008). Thus, it is perhaps unsurprising that a 20 kcal portion of rice could be seen to deliver the same amount of satiation as a 210 kcal beverage. The key finding is that the increase in expected satiation was equivalent regardless of whether the calories were delivered in a beverage or a solid food.

It is well known that prior experience plays an important role in shaping an individual's beliefs about the nutritive and satiating effects of a food. In regards to expected satiation, increased familiarity with eating a food has been shown to positively predict the amount of satiation it is expected to confer; an effect known as ‘expected satiation drift’ (Brunstrom et al., 2010; Ferriday, Rogers, Fay, Shakeshaft, & Brunstrom, 2011; Hardman, McCrickerd, & Brunstrom, 2011). With this is mind, we reasoned that participants who frequently consume sugar-containing beverages might be more familiar with their satiating effect and that this might result in higher estimates of expected satiation for meals paired with a sugar-containing drink, and greater magnitude of discrimination between the satiation of the caloric and non-caloric sweetened drinks. Experiment 2 provided support for this hypothesis, showing that participants who had a higher frequency of consuming sugar-containing beverages expected greater satiation from meals containing those beverages vs. sugar-free beverages. For now, it is unclear why these relationships were not also observed in Experiment 1 considering that both samples were similar in age, body weight, and history of soft drink consumption. However, we did broaden our measure of beverage familiarity to assess a wider variety of non-caloric and caloric sweetened beverages in Experiment 2—it is possible that this may have given us greater power to detect relationships between familiarity and expected satiation.

How these relationships between dietary intake and expected satiation come to be acquired remains to be determined. In Experiment 2, we found that total intake of sugar-sweetened beverages (i.e., fruit juices and non/-carbonated drinks) predicts greater expected satiation of meals containing sugar-containing beverages. This would seem to suggest that participants learn to associate different types of beverages with different postingestive outcomes/energy loads. However, participants' intake of Coca Cola was not related to their expected satiation of the meal that contained Coca Cola in either Experiment 1 (*r* = 0.04) or Experiment 2 (unreported). One explanation is that an individual's dietary experiences inform judgments of expected satiation across broad food categories (pastries, salads, pasta dishes, etc) more than being single-food or ‘product’ specific. However, this idea is speculative and remains to be scientifically tested.

The results of the present study must be interpreted within its limitations. One limitation is that we did not include a condition where the test meal was presented on its own, without a beverage. Adding a 500 ml bottle of water may have conferred no additional satiating effects to the meal (DellaValle, Roe, & Rolls, 2005; Rolls, Bell, & Thorwart, 1999), or it could have increased the overall expected satiation of the meal (Gray, French, Robinson, & Yeomans, 2003). A second limitation is that we did not control participants' food intake in the hours preceding the test. However, as this design relied on within-subjects comparisons, any baseline differences in appetite among our participants would not account for the differences among the beverage and snack conditions that were observed here.

A final limitation is that our comparison of the expected satiation of liquid versus solid foods is limited to two high-energy snack foods. We could not compare an exhaustive variety of liquid foods and solid foods in the present experiment and, thus, our conclusions about the relative expected satiation of liquids versus solids are restricted to the foods used here. However, using only two food items, we were able to demonstrate that beverages need not always differ in their expected satiation from solid foods. Future studies investigating the effects of food form might wish to expand their stimuli to test a range of different beverages and liquid foods to further explore this overlap with solid foods.

## Figures and Tables

**Table 1:** Relationships (Pearson's correlations) between beverage familiarity and the expected satiation of test meals.^a^

	*M (SD)*	Expected satiation of test meals				
Diet Coke	Orange Juice	Coca Cola	(Caloric – Non-caloric)	(Solid – Liquid)
LES intake	4.4 (2.3)	0.10	0.12	0.28*	0.42*	−0.14
SSB + Juice intake	10.4 (3.7)	0.14	0.23	0.27*	0.38*	−0.12

Intake of LES and SSB + Juice were coded according to frequency, with 1 = Less than once a month, 2 = 1–3 times per month, 3 = Once a week, 4 = 2–3 times per week, and 5 = Daily.

a Partial correlations between beverage familiarity and expected satiation after controlling for baseline appetite (hunger, fullness), baseline thirst, and item palatability (i.e., liking ratings collected for Coca-Cola, Diet Coke, Orange Juice, Haribo, and Peanuts). *p < 0.05.
